# Lifestyle factors, demographics and medications associated with depression risk in an international sample of people with multiple sclerosis

**DOI:** 10.1186/s12888-014-0327-3

**Published:** 2014-12-03

**Authors:** Keryn L Taylor, Emily J Hadgkiss, George A Jelinek, Tracey J Weiland, Naresh G Pereira, Claudia H Marck, Dania M van der Meer

**Affiliations:** Department of Psychiatry, St Vincent’s Hospital Melbourne, Victoria, VIC 3065 Australia; Department of Medicine, The University of Melbourne St Vincent’s Hospital, Melbourne, Victoria Australia; Emergency Practice Innovation Centre, St Vincent’s Hospital, Melbourne, Victoria Australia; Department of Epidemiology and Preventive Medicine, Monash University, Melbourne, Victoria Australia; Faculty of Medicine, Notre Dame University, Fremantle, Western Australia Australia

**Keywords:** Multiple sclerosis, Depression, Lifestyle, Survey

## Abstract

**Background:**

Depression is the most common co-morbidity for people with Multiple Sclerosis (MS); irrespective of disease severity, depression has the greatest impact on quality of life. An emerging paradigm in the treatment of depression is lifestyle medicine. There is significant potential to prevent and treat depression through modification of lifestyle risk factors for people with MS. This study sought to understand the association between lifestyle risk factors, medication and depression risk through the analysis of self-reported data from a large international sample of people with MS.

**Methods:**

This cross-sectional analysis recruited a total of 2459 participants via Web 2.0 platforms. Survey data included socio-demographics; a range of lifestyle risk factors; medication; disease variables and depression risk using the Patient Health Questionnaire-2 (PHQ-2).

**Results:**

In total approximately one fifth (19.3%) of our sample screened positive for depression (PHQ-2 score ≥3). Several demographic factors were significantly associated with this depression risk in bivariate analysis. Regression analyses showed that poor diet, low levels of exercise, obesity, smoking, marked social isolation and taking interferon were associated with greater depression risk. Participants who supplemented with omega 3s, particularly flaxseed oil, had frequent fish consumption, supplemented with vitamin D, meditated, and had moderate alcohol consumption had significantly reduced depression risk.

**Conclusions:**

This study demonstrates a significant association between modifiable lifestyle factors and depression risk. Planned longitudinal follow up may clarify causality. Clinicians and people with MS should be aware of the wide range of modifiable lifestyle factors that may reduce depression risk as part of a comprehensive secondary and tertiary preventive medical approach to managing MS.

## Background

Multiple sclerosis (MS) is a chronic autoimmune, inflammatory and demyelinating disease of the central nervous system. Symptoms can include motor and sensory deficits, ataxia, visual impairment, bladder and bowel incontinence, cognitive impairment, pain and fatigue. However for people with MS it is depression, irrespective of disease severity that has the greatest impact on quality of life [1].

Depression is the most common psychiatric illness and co-morbidity for people with MS, who are also at higher risk of suicide and self-harm than others in the population [2]. Severity of depression is a risk factor associated with suicide risk. For people with MS, the lifetime prevalence of a major depressive disorder is 50%, although an Australian study estimated an even higher rate of 67% [3]. The annual incidence is estimated to be 20% [4].

Although the high prevalence of depression in people with MS is widely acknowledged, depression is under-recognised and poorly treated [5]. People with MS who are depressed have increased use of outpatient and inpatient services, require comprehensive rehabilitative periods, and require more unsalaried care than those without depression [6]. The timely and effective treatment of depression for people with MS is vital. Treatment should be provided by mental health professionals in collaboration with general MS care [7].

A recent Cochrane review of pharmaceutical treatment of depression in MS failed to find any antidepressant medication that was significantly effective in treating depression in this patient group [8]. In fact a recent review of MS literature concluded there are no evidence based guidelines for either pharmacological or psychological treatments for people with MS and depression [9].

An emerging paradigm in the treatment of depression is lifestyle medicine. There is clear evidence that lifestyle factors are linked to the pathogenesis of mood disorders [10]. Many lifestyle factors are modifiable yet there is often little consideration of this treatment strategy, and pharmacological and psychological therapies remain the first line treatment choices [10]. A recent randomized controlled trial showed that modification of lifestyle factors (diet, sunlight exposure, exercise and sleep patterns) was an effective treatment strategy for depression [11]. Evidence-based recommendations can also be made for the use of mindfulness-based meditation as a treatment intervention for depression in people with MS [12]. There is strong evidence that smoking [13] and lack of social support are risk factors for depression [14,15]. The evidence for lifestyle medicine encourages an integrative approach whereby lifestyle modification is a routine part of prevention and treatment for depression. Lifestyle medicine for depression brings additional benefits to general health, particularly in reducing the likelihood of other high prevalence chronic western lifestyle related diseases such as cardiovascular disease and diabetes [10,13,16].

There appears to be significant potential to prevent and treat depression through modification of lifestyle risk factors for people with MS, although data are currently limited. In the Health Outcomes and Lifestyle Interventions in a Sample of People with Multiple Sclerosis (HOLISM) Study, we sought to understand the association between lifestyle risk factors amenable to modification and disease outcomes in general, through the analysis of self-reported data from a large international sample of people with MS. In this current study, part of the wider HOLISM research, we examined depression in particular, and its association with modifiable lifestyle risk factors and medication use, in order to better understand the potential for a secondary and tertiary preventive medical approach to managing depression in MS [17-20].

## Methods

### Participants and recruitment

The methodology for the HOLISM Study has previously been documented in detail [17]. In summary, participants were recruited via posting a link to the survey on Web 2.0 platforms that engaged people with MS. Participants completed a survey via SurveyMonkey® that examined their health and lifestyle behaviours, self-reported disability, disease activity, quality of life and depression, among other factors. Participants were eligible for the study if they were over 18 years of age and had been diagnosed with MS by a medical doctor. Participants were presented with an information page and selected “I agree” at the bottom of the page to provide consent before entering the survey. Ethics approval was granted by St Vincent’s Hospital Melbourne Human Research Ethics Committee (LRR 055/12).

### Data collection and tools used

For this study the survey data included items assessing the following: socio-demographics; diagnostic history; level of disability; co-morbidities; fatigue; and depression; as well as a range of lifestyle and health behaviours.

### Demographic data

Age, age at diagnosis, type of MS, gender, marital status, number of children, education level, employment status, weight and height were assessed and body mass index (BMI) was calculated by dividing weight (in kilograms) by height^2^ (in centimeters) and recoded into categories according to the World Health Organization (WHO) [21]. Those with BMI below 18.5 were categorized as underweight, those with BMI of 18.5 and up to 25 normal, BMI of 25 and up to 30 overweight and BMI of 30 and over obese.

### Depression

The Patient Health Questionnaire (PHQ-9) is a screening tool for depression, which has previously been validated in people with MS [22]. In our study we used the Patient Health Questionnaire depression module short version (PHQ-2). The PHQ-2 is scored in response to the question: ‘Over the past 2 weeks, how often have you been bothered by any of the following problems: Little interest or pleasure in doing things; Feeling down, depressed, or hopeless’ as follows: Not at all [0], Several days [1], More than half the days [2], Nearly every day [3]. The PHQ-2 is a shortened form of the PHQ-9 which has shown good criterion and construct validity. The PHQ-2 has a reported specificity of 92% and sensitivity of 83% for major depression with a score ≥3 [23]. This cut off was used in our analysis as a positive screen for depression risk. In addition, participants were asked to indicate whether they had a diagnosis of depression, whether they were receiving treatment for it, and whether depression limited their daily activities. The same questions were asked about an anxiety diagnosis. This question format was derived from the Self-Administered Comorbidity Questionnaire (SCQ) used to assess physical co-morbidities in the survey.

### Fatigue

The Fatigue Severity Scale (FSS) consists of nine fatigue-related statements rated on a seven-point scale from ‘disagree’ to ‘agree’ [24]. We used a mean score ≥4 to indicate clinically significant fatigue [25-27].

### Disability

The Patient-Determined Disease Steps (PDDS) is a self-reported surrogate tool to the Expanded Disability Status Scale (EDSS). It is an ordinal scale from O (normal) to 8 (bed bound) and has good correlation with the EDSS. It is also used as a practical tool to assess changes in people with MS across time [28] and has been used in numerous studies including the North American Research Committee on MS (NARCOMS) registry [29-31]. Due to a low number of cases in the ‘bed bound’ category, participants scoring 7 (‘wheelchair/scooter’) and 8 were grouped together to enable meaningful analysis. Analyses included people with all types of MS.

### Co-morbidities

We used the SCQ to assess co-morbidities in the absence of access to participants’ medical records. The SCQ determines if the co-morbidity limits activities and whether treatment is received. The SCQ has previously been used in a study of people with MS [32]. In our study, two arthritic co-morbidities were combined into one. All listed conditions were summed to determine an estimate of the number of co-morbidities each participant reported. Participants were then categorised as having: ‘none’, ‘1’, ‘2’, ‘3’, ‘4’, ‘5 or more’, co-morbidities.

### Dietary habits

For dietary assessment we used the Diet Habits Questionnaire (DHQ). The DHQ is a 24 item tool that has previously been used in an Australian cardiac population [33] and in the HOLISM study research on diet in MS [17-20]. We removed four items assessing salt use and alcohol intake. The remaining 20 items were scored from 1–5, giving rise to a summary score with a possible range of 20–100, with higher scores indicating more healthy dietary habits. Participants were grouped into quartiles, based on their summary score.

### Medication use

We used a researcher-generated list of disease modifying drugs (DMDs) and common MS drugs. We asked for specific details including past and current use and duration of use. For the purpose of analysis, participants were categorised according to whether they currently took one of the interferons (one of the currently licensed interferon beta preparations: Avonex, Rebif, Betaferon or Betaseron), a disease modifying drug other than an interferon, or “other” (including those who took a non disease modifying drug or who took none). Interferon use was analysed individually given its previously reported association with higher rates of depression [34].

### Vitamin D supplementation

We explored participants’ current vitamin D supplementation and calculated an average daily dosage. Participants were grouped as ‘none’, ‘< 5000 international units’ (IU) or ≥5000 IU.

### Omega-3 fatty acid supplementation

We assessed the type and average daily dose of omega-3 fatty acid supplementation used in the last 12 months. Types of omega-3 included flaxseed oil, fish oil, high strength fish oil, and ‘other’. Participants were grouped according to whether they took omega-3 supplementation (yes/no) and the type (‘flaxseed oil’, ‘fish oil and high strength fish oil’, ‘both’ or ‘none’). These comparisons were made based on findings from our previous work on omega-3s [20].

### Exercise

We used the International Physical Activity Questionnaire (IPAQ), which assesses the duration and frequency of vigorous and moderate physical activity, walking and sitting over the last 7 days. The IPAQ has been extensively validated, including in MS populations [35,36]. Participants were categorised as low active, moderate active, or high active, as defined by the IPAQ scoring instructions.

### Meditation

We asked participants how often they meditated on an average weekly basis. Response options were categorised for the purpose of analysis as ‘never’, ‘less than once per week’, ‘1–4 times per week’ or ‘5 or more times per week’.

### Social support

We used the Single Item Measure of Social Support (SIMSS) to determine how many people provided support to participants. It has previously been used in several studies involving cancer patients [37,38]. Response options were: none, 1 person, 2–5 people, 6–9 people, or 10 or more people. For the purpose of our study, the latter two categories were collapsed together.

### Data analysis

IBM SPSS Statistics 22.0 was used to analyse the data. Univariate analyses were performed and continuous data reported using mean (95% CI) or median (IQR), and categorical data using number and percentage. The denominator for each item varied due to variation in the number of participants completing each item, as per tabulated results.

Bivariate analyses were used to explore the relationship between socio-demographic factors, disease-specific variables, and the depression outcome measure (PHQ-2) to determine factors that should be included as covariates in regression modeling.

The complexity of interactions between the sociodemographic, health-related and lifestyle variables make it difficult to determine which lifestyle variables are uniquely associated with depression. Multivariate analysis examined a range of modifiable risk factors (‘exposure variables’, i.e. diet, exercise, vitamin D) and their association with screening positive for depression. Medication use could be regarded as a ‘modifiable risk factor’ for depression [34] and was therefore examined as an exposure variable.

Binary logistic regression was used to predict depressive risk (those scoring ≥3). For each modifiable risk factor individual models were derived adjusting for years since diagnosis, numbers of comorbidities, level of disability, clinically significant fatigue, age, gender and level of education. Type of MS was highly related with level of disability and we therefore chose to include only level of disability in the model. To maintain simplicity, we did not include employment in this model due to the number of categories within this variable. Because depression and fatigue have a complex relationship in people with MS, and fatigue may mediate depressive features [39], after controlling for fatigue as a potential confounder in multivariate analyses, we additionally compared models both with and without fatigue. There was negligible (up to approximately 10% in either direction) change to parameter estimates for all lifestyle variables when fatigue was removed from the model, and hence these results are not reported.

## Results

The demographics of this cohort (Table 1) have been described in detail in previous papers [17-20]. A high percentage (90.1%) of our sample answered the PHQ-2 screening questions for depression, Table 1 shows the demographics for the 2225 participants who completed the PHQ-2. The majority originated from the United States of America, Australia and the United Kingdom, with participants overall representing 54 different countries. Participants were mainly female (82.4%) and middle aged (median age 45 years). The majority were well educated holding a bachelor (36.5%) or post-graduate degree (23.5%), and working either full time (32.8%) or part time (21.3%), with almost a quarter retired due to disability. Most had a partner (74.8%) and children (68.9%). Participants had a median age of 37 years at diagnosis, on average had been recently diagnosed (45.2% within the last 5 years) and had mild disability (54.8% had PDDS scores 0–2). The majority (61.3%) had relapsing remitting MS.

**Table 1:** **Demographics and characteristics**

		**Median**	**IQR**
Age (years)		45	(38–53)
Years since diagnosis		6	(3–12)
Age at diagnosis (in years)		37	(30–45)
		N	%
Gender	Male	388/2201	17.6
	Female	1813/2201	82.4
Marital status	Married/cohabiting/partnered	1642/2194	74.8
	Single	304/2194	13.9
	Separated/Divorced/Widower	248/2194	11.3
Children	None	682/2196	31.1
	One or more	1514/2196	68.9
Education	No school/primary/secondary	533/2216	24.1
	Vocational training	355/2216	16.0
	Bachelor degree	808/2216	36.5
	Postgraduate	520/2216	23.5
Employment status	Part time	473/2220	21.3
	Retired due to disability	508/2220	22.9
	Work full time	729/2220	32.8
	Other*	510/2220	23.0
Type of MS	Benign	95/2217	4.3
	Relapsing-remitting	1359/2217	61.3
	Primary progressive	160/2217	7.2
	Secondary progressive	256/2217	11.5
	Progressive relapsing	46/2217	2.1
	Unsure/other	301/2217	13.6
Disability	Normal	714/2218	32.2
	Mild disability	342/2218	15.4
	Moderate disability	159/2218	7.2
	Gait disability	354/2218	16.0
	Early cane	245/2218	11.0
	Late cane	168/2218	7.6
	Bilateral support	135/2218	6.1
	Wheelchair/Scooter	98/2218	4.4
	Bedridden	3/2218	.1

*Includes stay at home parents, full time students, unemployed (seeking and not seeking employment), retired due to age and other. IQR, inter quartile range.

In total approximately one fifth (19.3%, N = 429) of our sample screened positive for depression (depression risk). Overall, 18.9% (N = 420) reported little interest or pleasure in doing things and 14.5% (N = 322) reported feeling down, depressed or hopeless, more than half the days or nearly every day. Nearly one third (30.9%, N = 686) of respondents reported depression as a current comorbidity and of those, 70.6% (N = 484) reported receiving treatment for the condition and 39.1% (N = 268) reported depression limiting their activities. Of the whole sample, 21.8% (N = 484) were taking a prescription medication for depression. Nearly 10% (9.6%, N = 47) of those taking prescription medication for depression stated that they did not have the condition.

Of those who screened positive for depression, 92.9% (N = 379) had a mean score of 4 or above on the FSS, indicating clinically significant fatigue.

### Demographic factors associated with depression risk

Regression analysis indicated that for every one year increase in age, participants had approximately 1.1% reduction in the odds of screening positive for depression (OR 0.989, 95%CI 0.979–0.999; p < 0.032). For every one year increase since diagnosis, participants had approximately 1.4% increase in the odds of screening positive for depression (OR 1.014, 95%CI 1.000–1.028; p = .049). For every one year increase at age of diagnosis, participants had a 2.0% decrease in the odds of screening positive for depression (OR 0.980, 95%CI 0.969–0.991; p < 0.001). Participants with a secondary school education and those with vocational training had around twice the odds of screening positive for depression of those with a postgraduate degree (Table 2). Participants who were separated, divorced or widower had 1.7 times greater odds of screening positive for depression than those married or co-habiting. Participants who were unemployed or retired due to disability had around twice the odds of screening positive for depression of those working full-time. Those with moderate or severe disability also had around twice the odds of screening positive for depression compared to those with mild disability. Type of MS was significantly associated with depression risk in that people with progressive relapsing MS were almost 2.5 times more likely to screen positive for depression than those with relapsing-remitting MS. There was a clearly increasing depression risk as the number of comorbidities increased and those with clinically significant fatigue were 9 times more likely to screen positive for depression. Participants’ gender and whether or not they had children was not significantly associated with depression risk (data not shown).

**Table 2:** **Odds ratios for risk of depression for demographic and disease factors**

**Variable**	**Groups**	**Positive depression screen^, N(%)**	***p***	***Unadjusted OR***	***p***
Education	Postgraduate degree	**70/ 520 (13.5)†**	**<0.001**	1.0	-
	Bachelor degree	**137 808/(17.0)†**		1.31 (0.96–1.79)	0.087
	Vocational training	84/ 355 (23.7)*		**1.99 (1.40–2.83)**	**<0.001**
	No school/primary/secondary	138/ 533 (25.9)*		**2.25 (1.64–3.09)**	**<0.001**
Marital status	Married/partnered/cohabitating	**285/ 1642 (17.4)†**	0.001	1.0	-
	Single	73/ 304 (24.0)*		**1.51 (1.12–2.02)**	**0.006**
	Separated/divorced/widow(er)	64/ 248 (25.8)*		**1.66 (1.21–2.26)**	**0.002**
Employment	Work full time	**119/ 729 (16.3)†**	<0.001	1.0	-
	Work part time	**66/ 473 (14.0)†**		0.83 (0.60–1.15)	0.266
	Stay at home parent/carer	**26/ 172 (15.1)†**		0.91 (0.58–1.45)	0.698
	Unemployed	46/167 (27.5)*		**1.95 (1.32–2.89)**	**0.001**
	Retired due to disability	150/ 508 (29.5)*		**2.15 (1.63–2.82)**	**<0.001**
	Retired due to age	**6/ 70 (8.6) †**		0.48 (0.20–1.14)	0.095
	Other (includes student)	**15/ 101 (14.9)†**		0.89 (0.50–1.60)	0.706
Disability	Mild	**169/ 1215 (13.9)†**	**<0.001**	1.0	-
	Moderate	199/ 767 (25.9)*		**2.17 (1.73–2.73)**	**<0.001**
	Severe	59/ 236 (25.0)*		**2.06 (1.74–2.89)**	**<0.001**
Type of MS	Relapsing-remitting	**252/ 1359 (18.5)†**	**0.015**	1.0	-
	Benign	10/ 95 (10.5)		0.52 (0.27–1.01)	0.053
	Primary progressive	35/ 160 (21.9)		1.23 (0.83–1.83)	0.309
	Secondary progressive	56/ 256 (21.9)		1.23 (0.89–1.71)	0.214
	Progressive relapsing	16/ 46 (34.8)*		**2.34 (1.26–4.36)**	**0.007**
	Unsure/other	58/ 301 (19.3)		1.05 (0.76–1.44)	0.770
Number of comorbidities	None	**53/ 737 (7.2)†**	**<0.001**	1.0	-
	One	**86/ 583 (14.8)†**		**2.23 (1.56–3.21)**	**<0.001**
	Two	110/ 452 (24.3)*		**4.15 (2.92–5.91)**	**<0.001**
	Three	103/ 259 (39.8)*		**8.52 (5.86–12.39)**	**<0.001**
	Four	40/ 110 (36.4)*		**7.38 (4.57–11.90)**	**<0.001**
	Five or more	36/ 80 (45.0)*		**10.56 (6.27–17.79)**	**<0.001**
Clinically significant fatigue	No	**29/ 732 (4.0)†**	**<0.001**	1.0	-
	Yes	379/ 1393 (27.2)*		**9.06 (6.14–13.38)**	**<0.001**

*denotes overrepresentation and † denotes underrepresentation ^ PHQ-2 score ≥3. Bold denotes statistically significant.

### Modifiable lifestyle factors

**Figure 1:**
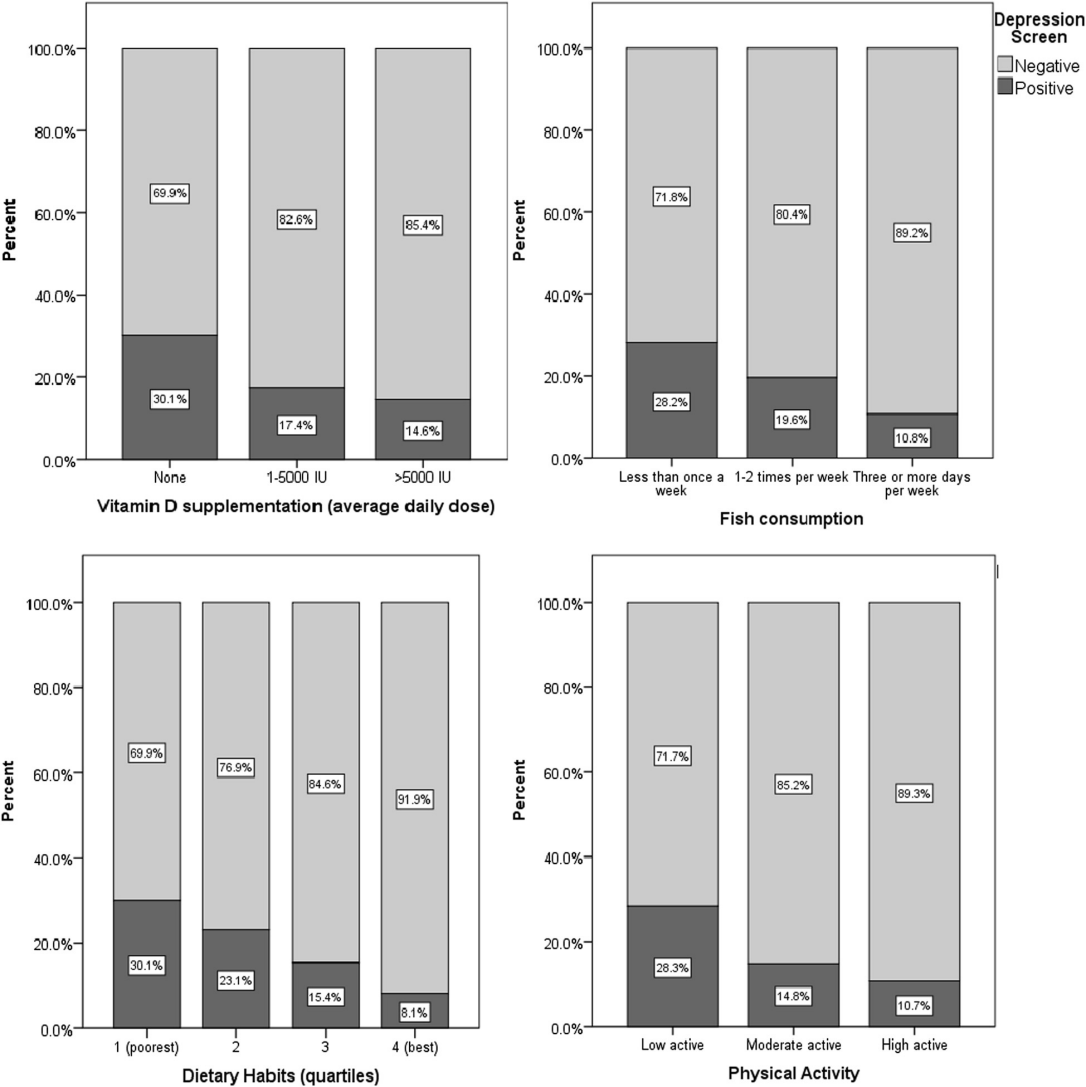
**Associations between lifestyle factors and depression screen.**

A number of modifiable lifestyle factors were significantly associated with depression risk in regression analyses adjusted for years since diagnosis, number of comorbidities, level of disability, clinically significant fatigue, age, gender, level of education and marital status. Figure 1 shows associations between depression screen and vitamin D supplementation, fish consumption, dietary habits and physical activity. Table 3 shows crude and adjusted odds ratios with 95% confidence intervals.

**Table 3:** **Crude and adjusted odds ratios, with 95% confidence intervals, for modifiable lifestyle risk factors and risk of depression**

		**Positive depression screen^, n/N(%)**	**p**	**Crude OR (95% CI)**	**A OR (95% CI)**
Smoking	Never	153/1072 (14.3)	<0.001	1.00 (reference)	1.00 (reference)
	Former	178/890 (20.0)		1.50 (1.19–1.90)**	1.54 (1.18–2.03)**
	Current	96/257 (37.4)		3.58 (2.64–4.86)***	2.29 (1.60–3.27)***
Alcohol	Low	310/1354 (22.9)	<0.001	1.00 (reference)	1.00 (reference)
	Moderate	113/834 (13.5)		0.53 (0.42–0.67)***	0.73 (0.55–0.96)*
	High	4/17 (23.5)		1.04 (0.34–3.20)	1.35 (0.38–4.74)
Exercise	Low	257/909 (28.3)	<0.001	1.00 (reference)	1.00 (reference)
	Moderate	104/701 (14.8)		0.44 (0.34–0.57)***	0.58 (0.43–0.78)***
	High	63/591 (10.7)		0.30 (0.23–0.41)***	0.51 (0.36–0.72)***
Vitamin D	None	117/389 (30.1)	<0.001	1.00 (reference)	1.00 (reference)
	1- 5000 IU	231/1324 (17.4)		0.49 (0.38–0.64)***	0.57 (0.43–0.77)***
	> 5000 IU	64/439 (14.6)		0.40 (0.28–0.56)***	0.47 (0.32–0.70)***
Omega 3	No	218/791 (27.6)	<0.001	1.00 (reference)	1.00 (reference)
	Yes	205/1420 (14.4)		0.44 (0.36–0.55)***	0.58 (0.45–0.75)***
Omega 3 type	None	218/791 (27.6)	<0.001	1.00 (reference)	1.00 (reference)
	Fish oil^	127/778 (16.3)		0.51 (0.40–0.66)***	0.64 (0.48–0.85)**
	Flaxseed oil	21/200 (10.5)		0.31 (0.19–0.50)***	0.48 (0.28–0.82)**
	Both fish oil and flaxseed oil	46/388 (11.9)		0.35 (0.25–0.50)***	0.49 (0.33–0.72)***
Fish consumption	Less than once per week	180/638 (28.2)	<0.001	1.00 (reference)	1.00 (reference)
	1–2 times per week	173/884 (19.6)		0.62 (0.49–0.79)***	0.71 (0.54–0.93)*
	3 or more days per week	75/696 (10.8)		0.31 (0.23–0.41)***	0.48 (0.34–0.67)***
Meditation	Never	241/1058 (22.8)	<0.001	1.00 (reference)	1.00 (reference)
	Less than once per week	99/493 (20.1)		0.85 (0.66–1.11)	0.86 (0.64–1.17)
	1–4 times per week	56/440 (12.7)		0.49 (0.36–0.68)***	0.52 (0.36–0.75)***
	5 or more times per week	29/227 (12.8)		0.50 (0.33–0.75)**	0.47 (0.29–0.75)**
Social support	6 or more	40/299 (13.4)	<0.001	1.00 (reference)	1.00 (reference)
	2–5	215/1316 (16.3)		1.26 (0.88–1.82)	1.11 (0.74–1.66)
	1	134/475 (28.2)		2.54 (1.73–3.75)***	2.42 (1.55–3.77)***
	None	34/111 (30.6)		2.86 (1.70–4.82)***	2.43 (1.32–4.47)**
Body Mass Index	Normal	176/1183 (14.9)	<0.001	1.00 (reference)	1.00 (reference)
	Underweight	16/92 (17.4)		1.21 (0.69–2.11)	0.97 (0.50–1.88)
	Overweight	113/504 (22.4)		1.65 (1.27–2.15)***	1.23 (0.91–1.67)
	Obese	116/421 (27.6)		2.18 (1.67–2.84)***	1.47 (1.08–2.01)*
Dietary habits	Upper (fourth) quartile	44/541 (8.1)	<0.001	1.00 (reference)	1.00 (reference)
	Third quartile	75/488 (15.4)		2.05 (1.38–3.04)***	1.65 (1.07–2.54)*
	Second quartile	116/502 (23.1)		3.39 (2.34–4.92)***	2.25 (1.48–3.41)***
	Lower (first) quartile	150/499 (30.1)		4.86 (3.38–6.98)***	2.73 (1.81–4.11)***
Medication	No DMD	187/1094 (17.1)	.004	1.00 (reference)	1.00 (reference)
	Interferon	104/424 (24.5)		1.58 (1.120–2.07)**	1.47 (1.07–2.02)*
	DMD not IFN	138/706 (19.5)		1.18 (0.92–1.05)	1.20 (0.90–1.60)

AOR: Adjusted odds ratios of screening positive for depression adjusted for years since diagnosis, number of comorbidities, level of disability, clinically significant fatigue, age, gender, marital status and level of education.

* < 0.05, ** < 0.01, *** < 0.001.

^ PHQ-2 score ≥3. Bold denotes statistically significant.

In summary, never smokers had the lowest odds of screening positive for depression, with increasing odds for former smokers, and greatest odds for current smokers. Participants who had moderate alcohol intake had significantly lower odds of screening positive for depression compared to participants with low alcohol intake. There was a dose-response effect with increasing levels of exercise associated with lower odds of screening positive for depression. Taking any vitamin D supplement was associated with lower odds of screening positive for depression, but taking at least 5,000 IU daily was associated with the greatest odds. Participants who supplemented with omega 3s had significantly reduced odds of screening positive for depression. Those supplementing with flaxseed oil had greater reduction in odds than those supplementing with fish oil. There was a clear dose-response effect for frequency of fish consumption, with most frequent fish consumers having the least odds of screening positive for depression. Meditation was associated with lower odds of screening positive for depression for those who meditated once or more per week. The odds of screening positive for depression increased with increasing social isolation but was only significant for those with very marked social isolation (none or one support person). Obese participants had significantly greater odds of screening positive for depression compared to those with a normal BMI. There was a clear dose-response effect for increasingly poor diet being associated with greater odds of screening positive for depression. Participants taking one of the interferons had significantly increased odds.

## Discussion

The prevalence of depression for people with MS is considerably higher than that of the general population [40] and higher than with other chronic illnesses [41,42] probably because of the multifactorial nature of depression in MS. While it may be reactive to the diagnosis of a chronic illness, depression in MS is also likely to be endogenous, partly based on the link with sclerotic pathology in specific neurological territories [43]. In a study controlling for disease-related variables and demographics, people with MS had three times the risk of depression compared to those with rheumatoid arthritis, suggesting that these differences could in part be attributed to the biological impact of the disease process in MS. [41] Additionally there is a strong link between genetics and depression in the general population when people with MS are excluded; however, for people with MS who have depression there is not a clear genetic basis, adding further weight to the role of other contributing factors [44,45].

Depression symptoms increase during a relapse, however they do not typically resolve following recovery but may become persistent [46]. Depression can also exacerbate or be implicated in other symptoms of MS including fatigue (2), cognitive processing and working memory [47,48], as well as the experience of pain symptoms (4). In our study however, fatigue did not appear to mediate the effects of depression, and its inclusion in regression modelling had little impact on the observed associations.

The high prevalence of depression in people with MS and its likely reactive and endogenous mechanisms are important for two reasons. Firstly they highlight the need for screening, assessment and treatment of depression in people with MS. Secondly, by drawing attention to the direct disease impact on depression, patients may more readily be able to overcome the self-stigma of depression and seek vital treatment [41].

It is important to alleviate depressive symptoms to improve quality of life but also to avoid worsening of physical health outcomes. A growing body of evidence highlights the pro-inflammatory immunological changes seen in depression and the link to disease activity in MS. Depression increases pro-inflammatory cytokines and inflammation, thereby worsening MS [48] and is associated with an increase in interferon secretion by T lymphocytes [49]. Several of the lifestyle behaviours found to be associated with depression risk in our study have also been shown to modulate inflammation, such as smoking, exercise and meditation [50-52].

It is important to prevent and treat depression to optimize adherence to disease modifying therapies [4,53] and to monitor people with MS for the potential iatrogenic effects of disease modifying medication leading to depression (4).

In this context of negative mental, physical and quality of life consequences of depression, it is important for clinicians and researchers to better understand the lifestyle and medication risk factors that may lead to depression in people with MS, and that may be amenable to modification. Through a comprehensive investigation of modifiable risk factors for depression in a large international sample of people with MS, our study is an important addition to the literature. We have shown strong and highly significant associations between modifiable lifestyle factors and medication use and depression risk, providing valuable information for clinicians and people with MS alike about the key factors that may be modified to reduce depression risk.

Demographic and disease variables significantly associated with higher odds of screening positive for depression were lower educational status, unemployment or retirement due to disability and being separated or divorced. Those who were younger, diagnosed for longer, with more relapses over the previous year, more severe disability, with a progressive relapsing type of MS, and a higher number of comorbidities also had higher odds of screening positive for depression. These findings are consistent with recent research and underscore the importance of social interventions for people with MS such as support to maintain employment [54].

In line with previous research in MS and other neurological disorders [14,15], our data showed a very strong relationship between less social support and increased odds of screening positive for depression. Participants with no or little social support had nearly 2.5 times the odds compared to those with high levels of support; this was one of the strongest associations of all the variables we studied in regression analysis. Self-management programs that offer the opportunity for group support may help reduce depression risk in people with MS, as previously shown in follow up studies demonstrating improved mental health after residential group programs [55,56].

Diet had a very strong association with depression risk, both clinically and statistically, with a clear dose-response effect of increasing odds of screening positive for depression with worsening diet. Those with very poor diet had a nearly threefold increase in odds. Our data are consistent with systematic reviews of observational and prospective studies showing that healthy dietary patterns positively influence depression in the general population [57,58]. Given other beneficial effects of healthy diet in MS [18] and generally [59], it is important for clinicians to provide dietary advice as a key component of depression risk management in MS.

As in the general population [60], smokers in our study had approximately two and a half times greater odds of screening positive for depression than non-smokers, while former smokers also had higher odds compared to participants who had never smoked. This finding is also consistent with the pattern observed for health-related quality of life (HRQOL), including emotional well-being and mental health composite scores, seen in people with MS [19] and in the general population [61]. The proposed immunomodulatory or neurotoxic effects of smoking [62] may share common aetiological pathways in depression for people with MS [63].

Exercise was strongly associated with depression risk, again with a dose-response effect. Participants with high levels of exercise had half the odds of screening positive for depression while those with moderate levels had a lesser reduction. People with MS who regularly participate in physical activity are more likely to have fewer depressive symptoms and to have reducing depressive symptoms across time [64,65]. Again, given the generally beneficial effects of exercise, and particularly for those people with MS [65], clinicians should offer this advice to people with MS.

Similarly, the odds of screening positive for depression was roughly halved in participants supplementing with omega 3 fatty acids, with a greater reduction for flaxseed oil than for those supplementing with fish oil alone or in combination with flaxseed oil, in line with our previously reported data on relapse rate reductions [20]. People consuming fish more frequently had similar reductions in the odds of screening positive for depression, with a dose-response effect, again in line with previously reported benefits to other health outcomes in people with MS [20]. Epidemiological research indicates that low omega 3 fatty acid consumption is associated with higher rates of depression in the general population [66]. Animal and clinical studies show that omega 3s have strong anti-inflammatory properties and may be effective in modulating inflammatory diseases such as MS and the associated depression [67]. Our data strongly support a role for omega 3 supplementation in reducing depression risk in people with MS, in addition to its clear association with reduced risk of relapse [20].

Vitamin D supplementation in combination with fluoxetine was more effective than fluoxetine alone in reducing depressive symptoms in a randomized controlled trial of patients with depression in the general population [68]. For people with MS, low vitamin D is significantly associated with depressive symptoms [69]. Our data support a role for vitamin D supplementation in reducing depression risk for people with MS, with a halving of odds of screening positive for depression for participants taking more than 5000 IU of vitamin D daily, and a dose-response effect. A dose of at least 5000 IU daily has previously been suggested as an optimal dose for people with MS [70].

The relationship between obesity and depression is bi-directional with inflammation playing an intermediary role. Adipocytes secrete tumour necrosis factor alpha and interleukin 6 leading to increased leukocytes and chronic inflammation. Inflammation in turn is linked to many lifestyle-related diseases such as MS, diabetes and cardiovascular disease [71]. Obese participants in our study had 1.5 times greater odds of screening positive for depression than those with normal BMI. Weight loss is potentially an effective intervention to improve both mental and physical health, particularly for people with MS [72], and clinicians treating people with MS should incorporate this into an overall preventive management plan.

Our study failed to detect any increased odds of screening positive for depression associated with alcohol intake in people with MS, although may have been limited in power because of small numbers consuming alcohol in large amounts. There was a suggestion of a U-shaped pattern of association between level of alcohol use and depression risk, with moderate alcohol intake associated with almost 1.5 times reduced odds of screening positive for depression compared to low levels of alcohol use. This pattern is in line with data observed for HRQOL in people with MS [19] and in mortality data for other chronic illnesses [45] supporting the external validity of our study’s self-reported data; we did not detect any signal suggesting significant health risks for people with MS drinking moderate amounts of alcohol.

Recent meta-analysis of meditation programs shows an improvement in depression, HRQOL mental health composite scores and psychological stress in the general population [73]. Consistent findings from a randomized controlled trial showed that mindfulness based interventions improved depression and HRQOL at six months follow up in people with MS, while control subjects had a worsening of symptoms [12,74]. In line with these studies, we have shown a halving of the odds of screening positive for depression in people with MS who meditated once or more per week. Numerous neurobiological mechanisms underpin the benefits of meditation for mental health, including attention and emotional regulation, cognitive re-evaluation and body awareness. Mindfulness meditation also holds benefit for physical health for people with MS by improving immune function and reducing inflammation [75].

Finally, while the interferons have been shown in randomized controlled trials to reduce relapse rates modestly, there has been concern about their side effects. Interferons may worsen depressive symptoms in people with and without pre-existing depression [34]. An uncommon, yet serious adverse effect of interferon treatment for people with MS is severe depression with active suicidal ideation and suicide attempts [34]. Our finding that participants taking interferon had nearly 50% increased odds of screening positive for depression, while those taking other disease-modifying medications did not appear to be at heightened risk of depression. This highlights the need for clinicians to carefully consider, assess and monitor patients’ mental health when suggesting medication choices, and carefully communicating potential risks to their patients.

### Limitations

Overall 19.3% of our study’s participants screened positive for depression. Current literature suggests the annual incidence of depression for people with MS is approximately 20%. (5) Overall, 31.7% of our study’s participants reported depression as a current co-morbidity. The exact proportion meeting diagnostic criteria for depression within this study population remains unknown. Our sample may be biased however, as participants in our online survey were more likely to be engaged in active coping strategies positively improving their mental health and thereby lowering their depression risk compared to the general population of people with MS who frequently adopt avoidance as a coping strategy [64].

The PHQ-2 is well validated and rigorous and likely detects those with more severe symptomatology. Although the PHQ-2 is a short screening tool, the use of two questions about low mood and anhedonia is a simple and time-efficient screening tool for depression in MS that can be easily incorporated into routine care [76].

Reverse causality may contribute to these associations as depression may result in less healthy behaviours, such as being sedentary, eating badly, and smoking, perhaps through low motivation. However, the dose-response effect for many of the lifestyle variables, and the biological plausibility of the associations, reduce the likelihood of reverse causality playing a major role in these findings. Planned longitudinal follow up of participants may help clarify causality.

Participants self-reported data so the potential for measurement error such as recall bias exists, as well as potential for this error to be correlated between the exposures and outcomes (dependent misclassification) thus biasing results. The degree to which this is a problem in our data is not known.

We analysed all types of MS together and there may be differences in the associations of modifiable risk factors within subtypes of MS but the smaller sample sizes within each subtype and the number of variables controlled for in the multivariate analysis would limit the reliability of such results.

Our data may be limited as participants required the cognitive ability to complete the comprehensive survey and also to have access to the internet. The data can however be widely generalized given that our sample is large and geographically diverse and includes people with all types of MS.

## Conclusion

This study demonstrates a strong clinically and statistically significant association between modifiable lifestyle factors and risk of depression. Diet, smoking, exercise, omega 3 supplementation, (particularly flaxseed oil), fish consumption, social support, vitamin D supplementation, BMI, alcohol intake, meditation and choice of medication are important modifiable factors in depression risk for people with MS. It is important for clinicians and people with MS to be aware of the wide range of modifiable lifestyle factors that may reduce depression risk as part of a comprehensive secondary and tertiary preventive medical approach to managing MS.

## References

[1] D’Alisa S, Miscio G, Baudo S, Simone A, Tesio L, Mauro A (2006). Depression is the main determinant of quality of life in multiple sclerosis: a classification-regression (CART) study. Disabil Rehabil.

[2] Pompili M, Forte A, Palermo M, Stefani H, Lamis DA, Serafini G, Amore M, Girardi P (2012). Suicide risk in multiple sclerosis: a systematic review of current literature. J Psychosom Res.

[3] Khan F, McPhail T, Brand C, Turner-Stokes L, Kilpatrick T (2006). Multiple sclerosis: disability profile and quality of life in an Australian community cohort. Int J Rehabil Res.

[4] Jose Sa M (2008). Psychological aspects of multiple sclerosis. Clin Neurol Neurosurg.

[5] Kanner AM, Barry JJ (2003). The impact of mood disorders in neurological diseases: should neurologists be concerned?. Epilepsy Behav.

[6] Ytterberg C, Lundqvist S, Johansson S (2013). Use of health services in people with multiple sclerosis with and without depressive symptoms: a two-year prospective study. BMC Health Serv Res.

[7] Minden SL, Ding L, Cleary PD, Frankel D, Glanz BI, Healy BC, Rintell DJ (2013). Improving the quality of mental health care in multiple sclerosis. J Neurol Sci.

[8] Koch MW, Glazenborg A, Uyttenboogaart M, Mostert J, De Keyser J (2011). Pharmacologic treatment of depression in multiple sclerosis. Cochrane Database Syst Rev.

[9] Fragoso YD, Adoni T, Anacleto A, Da Gama PD, Goncalves MV, Matta AP, Parolin MF (2014). Recommendations on diagnosis and treatment of depression in patients with multiple sclerosis. Pract Neurol.

[10] Sarris J, O’Neil A, Coulson CE, Schweitzer I, Berk M (2014). Lifestyle medicine for depression. BMC Psychiatry.

[11] García-Toro M, Ibarra O, Gili M, Serrano MJ, Oliván B, Vicens E, Roca M (2012). Four hygienic-dietary recommendations as add-on treatment in depression: a randomized-controlled trial. J Affect Disord.

[12] Grossman P, Kappos L, Gensicke H, D’Souza M, Mohr DC, Penner IK, Steiner C (2010). MS quality of life, depression, and fatigue improve after mindfulness training: a randomized trial. Neurology.

[13] Jacka FN, Mykletun A, Berk M (2012). Moving towards a population health approach to the primary prevention of common mental disorders. BMC Med.

[14] Jensen MP, Smith AE, Bombardier CH, Yorkston KM, Miro J, Molton IR (2014). Social support, depression, and physical disability: age and diagnostic group effects. Disabil Health J.

[15] Vargas GA, Arnett PA (2010). Positive everyday experiences interact with social support to predict depression in multiple sclerosis. J Int Neuropsychol Soc.

[16] Stuifbergen AK, Becker H (2001). Health promotion practices in women with multiple sclerosis: increasing quality and years of healthy life. Physical Med Rehabil Clin North Am.

[17] Hadgkiss EJ, Jelinek GA, Weiland TJ, Pereira NG, Marck CH, Van Der Meer DM (2013). Methodology of an international study of people with multiple sclerosis recruited through Web 2.0 platforms: demographics, lifestyle, and disease characteristics. Neurol Res Int.

[18] Hadgkiss EJ, Jelinek GA, Weiland TJ, Pereira NG, Marck CH, Van Der Meer DM: **The association of diet with quality of life, disability, and relapse rate in an international sample of people with multiple sclerosis.** *Nutr Neurosci* 2014:1–13 10.1179/1476830514Y.0000000117 PMC4485697 24628020

[19] Weiland TJ, Hadgkiss EJ, Jelinek GA, Pereira NG, Marck CH, van der Meer DM (2014). The association of alcohol consumption and smoking with quality of life, disability and disease activity in an international sample of people with multiple sclerosis. J Neurol Sci.

[20] Jelinek GA, Hadgkiss EJ, Weiland TJ, Pereira NG, Marck CH, Van Der Meer DM (2013). Association of fish consumption and omega 3 supplementation with quality of life, disability and disease activity in an international cohort of people with multiple sclerosis. Int J Neurosci.

[21] **Global Database Of Body Mass Index. BMI Classification** [http://apps.who.int/bmi/index.jsp?introPage=intro_3.html]

[22] Sjonnesen K, Berzins S, Fiest KM AGMB, Metz LM, Thombs BD, Patten SB (2012). Evaluation of the 9-item Patient Health Questionnaire (PHQ-9) as an assessment instrument for symptoms of depression in patients with multiple sclerosis. Postgrad Med.

[23] Kroenke K, Spitzer RL, Williams JB (2003). The Patient Health Questionnaire-2: validity of a two-item depression screener. Med Care.

[24] Krupp LB, LaRocca NG, Muir-Nash J, Steinberg AD (1989). The fatigue severity scale. Application to patients with multiple sclerosis and systemic lupus erythematosus. Arch Neurol.

[25] Lerdal A, Celius EG, Moum T (2003). Fatigue and its association with sociodemographic variables among multiple sclerosis patients. Mult Scler.

[26] Smedal T, Beiske AG, Glad SB, Myhr KM, Aarseth JH, Svensson E, Gjelsvik B, Strand LI (2011). Fatigue in multiple sclerosis: associations with health-related quality of life and physical performance. Eur J Neurol.

[27] Marrie RA, Cutter G, Tyry T, Hadjimichael O, Campagnolo D, Vollmer T (2005). Validation of the NARCOMS registry: fatigue assessment. Mult Scler.

[28] Hohol MJ, Orav EJ, Weiner HL (1995). Disease steps in multiple sclerosis: a simple approach to evaluate disease progression. Neurology.

[29] Marrie RA, Cutter G, Tyry T, Hadjimichael O, Campagnolo D, Vollmer T (2005). Changes in the ascertainment of multiple sclerosis. Neurology.

[30] Marrie RA, Cutter G, Tyry T, Vollmer T, Campagnolo D (2006). Does multiple sclerosis-associated disability differ between races?. Neurology.

[31] Hadjimichael O, Vollmer T, Oleen-Burkey M, North American Research Committee on Multiple Sclerosis (2008). Fatigue characteristics in multiple sclerosis: the North American Research Committee on Multiple Sclerosis (NARCOMS) survey. Health Q Life Outcomes.

[32] Holper L, Coenen M, Weise A, Stucki G, Cieza A, Kesselring J (2010). Characterization of functioning in multiple sclerosis using the ICF. J Neurol.

[33] McKellar S, Horsley P, Chambers R, Pullen M, Vendersee P, Clarke C, Callum H, Bauer J (2008). Development of the diet habits questionnaire for use in cardiac rehabilitation. Aust Primary Health.

[34] Fragoso YD, Frota ER, Lopes JS, Noal JS, Giacomo MC, Gomes S, Goncalves MV, da Gama PD, Finkelsztejn A (2010). Severe depression, suicide attempts, and ideation during the use of interferon beta by patients with multiple sclerosis. Clin Neuropharmacol.

[35] Craig CL, Marshall AL, Sjostrom M, Bauman AE, Booth ML, Ainsworth BE, Pratt M, Ekelund U, Yngve A, Sallis JF, Oja P (2003). International physical activity questionnaire: 12-country reliability and validity. Med Sci Sports Exerc.

[36] Weikert M, Motl RW, Suh Y, McAuley E, Wynn D (2010). Accelerometry in persons with multiple sclerosis: measurement of physical activity or walking mobility?. J Neurol Sci.

[37] Reavley N, Pallant JF, Sali A (2009). Evaluation of the effects of a psychosocial intervention on mood, coping, and quality of life in cancer patients. Integr Cancer Ther.

[38] Koopman C, Nouriani B, Erickson V, Anupindi R, Butler LD, Bachmann MH, Sephton SE, Spiegel D (2002). Sleep disturbances in women with metastatic breast cancer. Breast J.

[39] Induruwa I, Constantinescu CS, Gran B (2012). Fatigue in multiple sclerosis - a brief review. J Neurol Sci.

[40] Lebrun C, Cohen M (2009). [Depression in multiple sclerosis]. Rev Neurol.

[41] Holden K, Isaac CL (2011). Depression in multiple sclerosis: reactive or endogenous?. Clin Neuropsychol.

[42] Heesen C, Kopke S, Kasper J, Poettgen J, Tallner A, Mohr DC, Gold SM (2012). Behavioral interventions in multiple sclerosis: a biopsychosocial perspective. Expert Rev.

[43] Vattakatuchery JJ, Rickards H, Cavanna AE (2011). Pathogenic mechanisms of depression in multiple sclerosis. J Neuropsychiatry Clin Neurosci.

[44] Sadovnick AD, Remick RA, Allen J, Swartz E, Yee IM, Eisen K, Farquhar R, Hashimoto SA, Hooge J, Kastrukoff LF, Morrison W, Nelson J, Oger J, Paty DW (1996). Depression and multiple sclerosis. Neurology.

[45] D’Hooghe MB, De Keyser J (2012). Associations of alcohol consumption with clinical and MRI measures in multiple sclerosis. Expert Rev Neurother.

[46] Moore P, Hirst C, Harding KE, Clarkson H, Pickersgill TP, Robertson NP (2012). Multiple sclerosis relapses and depression. J Psychosom Res.

[47] Feinstein A (2006). Mood disorders in multiple sclerosis and the effects on cognition. J Neurol Sci.

[48] Gold SM, Irwin MR (2009). Depression and immunity: inflammation and depressive symptoms in multiple sclerosis. Immunol Allergy Clin North Am.

[49] Pokryszko-Dragan A, Frydecka I, Kosmaczewska A, Ciszak L, Bilinska M, Gruszka E, Podemski R, Frydecka D (2012). Stimulated peripheral production of interferon-gamma is related to fatigue and depression in multiple sclerosis. Clin Neurol Neurosurg.

[50] Csiszar A, Podlutsky A, Wolin MS, Losonczy G, Pacher P, Ungvari Z (2009). Oxidative stress and accelerated vascular aging: implications for cigarette smoking. Front Biosci (Landmark Ed).

[51] Golzari Z, Shabkhiz F, Soudi S, Kordi MR, Hashemi SM (2010). Combined exercise training reduces IFN-gamma and IL-17 levels in the plasma and the supernatant of peripheral blood mononuclear cells in women with multiple sclerosis. Int Immunopharmacol.

[52] Levin AB, Hadgkiss EJ, Weiland TJ, Jelinek GA (2014). Meditation as an adjunct to the management of multiple sclerosis. Neurol Res Int.

[53] Saunders C, Caon C, Smrtka J, Shoemaker J (2010). Factors that influence adherence and strategies to maintain adherence to injected therapies for patients with multiple sclerosis. J Neurosci Nurs.

[54] Ensari I, Motl RW, McAuley E, Mullen SP, Feinstein A (2014). Patterns and predictors of naturally occurring change in depressive symptoms over a 30-month period in multiple sclerosis. Mult Scler.

[55] Hadgkiss EJ, Jelinek GA, Weiland TJ, Rumbold G, Mackinlay CA, Gutbrod S, Gawler I (2013). Health-related quality of life outcomes at 1 and 5 years after a residential retreat promoting lifestyle modification for people with multiple sclerosis. Neurol Sci.

[56] Li MP, Jelinek GA, Weiland TJ, Mackinlay CA, Dye S, Gawler I (2010). Effect of a residential retreat promoting lifestyle modifications on health-related quality of life in people with multiple sclerosis. Q Primary Care.

[57] Rahe C, Unrath M, Berger K (2014). Dietary patterns and the risk of depression in adults: a systematic review of observational studies. Eur J Nutr.

[58] Sanhueza C, Ryan L, Foxcroft DR (2013). Diet and the risk of unipolar depression in adults: systematic review of cohort studies. J Human Nutri Diet Off J British Dietetic Assoc.

[59] Sofi F, Macchi C, Abbate R, Gensini GF, Casini A (2013). Mediterranean diet and health status: an updated meta-analysis and a proposal for a literature-based adherence score. Public Health Nutr.

[60] Park S, Romer D, Lim S (2013). Does smoking initiation in adolescence increase risk for depression across the lifespan? Evidence from the South korean national health and nutrition examination survey. J Addict Nurs.

[61] Heikkinen H, Jallinoja P, Saarni SI, Patja K (2008). The impact of smoking on health-related and overall quality of life: a general population survey in Finland. Nicotine Tob Res Off J Soc Res Nicotine Tob.

[62] Kalra R, Singh SP, Savage SM, Finch GL, Sopori ML (2000). Effects of cigarette smoke on immune response: chronic exposure to cigarette smoke impairs antigen-mediated signaling in T cells and depletes IP3-sensitive Ca(2+) stores. J Pharmacol Exp Therapeutics.

[63] Edwards AC, Kendler KS (2012). A twin study of depression and nicotine dependence: shared liability or causal relationship?. J Affect Disord.

[64] Goretti B, Portaccio E, Zipoli V, Hakiki B, Siracusa G, Sorbi S, Amato MP (2009). Coping strategies, psychological variables and their relationship with quality of life in multiple sclerosis. Neurol Sci Off J Italian Neurol Soc Italian Soc Clin Neurophysiol.

[65] Stroud NM, Minahan CL (2009). The impact of regular physical activity on fatigue, depression and quality of life in persons with multiple sclerosis. Health Q Life Outcomes.

[66] Hibbeln JR, Salem N (1995). Dietary polyunsaturated fatty acids and depression: when cholesterol does not satisfy. Am J Clin Nutr.

[67] Simopoulos AP (2002). Omega-3 fatty acids in inflammation and autoimmune diseases. J Am Coll Nutr.

[68] Khoraminya N, Tehrani-Doost M, Jazayeri S, Hosseini A, Djazayery A (2013). Therapeutic effects of vitamin D as adjunctive therapy to fluoxetine in patients with major depressive disorder. Aust New Zealand J Psychiatry.

[69] Ashtari F, Ajalli M, Shaygannejad V, Akbari M, Hovsepian S (2013). The relation between Vitamin D status with fatigue and depressive symptoms of multiple sclerosis. J Res Med Sci Off J Isfahan Univ Med Sci.

[70] Brum DG, Comini-Frota ER, Vasconcelos CC, Dias-Tosta E (2014). Supplementation and therapeutic use of vitamin D in patients with multiple sclerosis: consensus of the Scientific Department of Neuroimmunology of the Brazilian Academy of Neurology. Arq Neuropsiquiatr.

[71] Shelton RC, Miller AH (2010). Eating ourselves to death (and despair): the contribution of adiposity and inflammation to depression. Prog Neurobiol.

[72] Lopresti AL, Drummond PD (2013). Obesity and psychiatric disorders: commonalities in dysregulated biological pathways and their implications for treatment. Prog Neuro-Psychopharmacol Biol Psychiatry.

[73] Goyal M, Singh S, Sibinga EM, Gould NF, Rowland-Seymour A, Sharma R, Berger Z, Sleicher D, Maron DD, Shihab HM, Ranasinghe PD, Linn S, Saha S, Bass EB, Haythornthwaite JA (2014). Meditation programs for psychological stress and well-being: a systematic review and meta-analysis. JAMA Inter Med.

[74] Tavee J, Stone L (2010). Healing the mind: meditation and multiple sclerosis. Neurology.

[75] Ngo TL (2013). Review of the effects of mindfulness meditation on mental and physical health and its mechanisms of action. Sante Mentale au Quebec.

[76] Fragoso YD (2014). Recommendations on diagnosis and treatment of depression in patients with multiple sclerosis. Pract Neurol.

